# Identification of Young High-Functioning Autism Individuals Based on Functional Connectome Using Graph Isomorphism Network: A Pilot Study

**DOI:** 10.3390/brainsci12070883

**Published:** 2022-07-05

**Authors:** Sihong Yang, Dezhi Jin, Jun Liu, Ye He

**Affiliations:** School of Artificial Intelligence, Beijing University of Posts and Telecommunications, Beijing 100876, China; yangsihong@bupt.edu.cn (S.Y.); dzjin@bupt.edu.cn (D.J.); liujun@bupt.edu.cn (J.L.)

**Keywords:** autism spectrum disorder, functional connectivity, deep learning, graph isomorphism network

## Abstract

Accumulated studies have determined the changes in functional connectivity in autism spectrum disorder (ASD) and spurred the application of machine learning for classifying ASD. Graph Neural Network provides a new method for network analysis in brain disorders to identify the underlying network features associated with functional deficits. Here, we proposed an improved model of Graph Isomorphism Network (GIN) that implements the Weisfeiler-Lehman (WL) graph isomorphism test to learn the graph features while taking into account the importance of each node in the classification to improve the interpretability of the algorithm. We applied the proposed method on multisite datasets of resting-state functional connectome from Autism Brain Imaging Data Exchange (ABIDE) after stringent quality control. The proposed method outperformed other commonly used classification methods on five different evaluation metrics. We also identified salient ROIs in visual and frontoparietal control networks, which could provide potential neuroimaging biomarkers for ASD identification.

## 1. Introduction

Autism Spectrum Disorder (ASD) is one of the major neurodevelopmental disorders characterized by deficits in social communication and restrictive/repetitive behaviors and interests [1]. At present, the diagnosis of ASD is mainly based on behavioral symptoms evaluated by clinical experts. The lack of biomarkers makes the diagnosis more challenging due to the heterogeneity of ASD; hundreds of genes have been found to be related to ASD [2,3]. Finding the biomarkers of ASD and making accurate diagnosis have been crucial problems that need to be solved.

Brain function depends on connections between brain regions [4]. Network analysis of brain structure and function have confirmed the general hierarchy principle of connectome organization [5]. Recent studies have shown that the peripheral networks of sensory and motor areas have more locally clustered connections [6], and the richly connected cerebral cortex consists of a selective set of frontoparietal hubs [7] that are involved in a wide range of behavioral and cognitive tasks [8]. Neuroanatomy and brain imaging provide support for changes at the level of the brain network throughout neural development [5]. Functional magnetic resonance imaging (fMRI) provides a non-invasive way to measure macroscopic functional connections [5]. fMRI measures the hemodynamic changes caused by the activity of neurons in the brain [9], which has been widely used in the study of brain dysfunction. It can capture the interactions between brain regions, which is called functional connectivity (FC) [10,11]. Studies using fMRI have shown the disorder is associated with altered connections [12,13]. fMRI provides a promising way to facilitate diagnosis.

Altered FC from resting-state fMRI (rs-fMRI) has been found in many previous studies of ASD in the past decades [14,15]. Hong et al. (2019) analyzed the cortex-wide functional connectomes and found perturbed functional gradients in autism, showing reduced functional distance between transmodal and unimodal regions [5]. Zhou et al. (2019) found SHANK3 mutant rhesus monkeys with autism-related symptoms showed abnormal connections in brain function, particularly inadequate long connections between the default mode network, including between the posterior cingulate gyrus, medial prefrontal cortex (mPFC) and motor areas [16]. Kim et al. (2022) proposed that ASD patients showed higher mPFC-amygdala functional connectivity at static and dynamic levels than the control group, and the connectivity was specific [17]. Fernandez et al. (2019) proposed that inadequate connectivity was the root cause of behavioral defects and may be the cause of brain diseases such as autism or schizophrenia [18]. These findings show that ASD is associated with widely spread brain regions, which infers that it is better to explore the biomarkers at a global network level rather than local regions.

Recently, this method has been shown to be effective in identifying autism in a control group using approaches from machine learning [19,20,21]. Plitt et al. (2015) achieved high accuracy in identifying ASD from the control group at a single site by linear SVM [22]. More deep learning techniques such as deep neural network (DNN) and 3D Convolutional Neural Networks have been applied to analyzing neuroimaging data and demonstrated good performance [23,24]. Graph Neural Networks (GNNs) is a state-of-the-art deep learning method for graph analysis. As the whole brain can be taken as a network containing connections between different brain regions, which is also called brain connectome [25]. The methods from graph theory have been widely used in analyzing the brain network. Recently GNNs and other graph analysis methods of deep learning have been applied to analyzing brain networks, especially used for the classification of brain disorders. Ktena et al. (2018) was the first to apply GCN to the functional brain network of ASD [26]. They proposed a Siamese graph convolutional neural network (s-GCN) in a supervised setting to determine the similarity metric of the graph. The proposed framework took into consideration the graph structure for the evaluation of the similarities between a pair of graphs. It employed spectral graph convolutions that allowed the generalization of traditional convolutions to irregular graphs and operated in the spectral domain of graphs. Parisot et al. (2017) introduced Graph Convolutional Networks (GCN) to brain analysis in populations, combining imaging and non-imaging data [27]. The sample population was represented as a sparse graph. Each node of the graph corresponded to the image feature vector of a single sample, and the edge weights were associated with the phenotypic data. The structure was used to train the GCN model on a partial label graph to infer the categories of unlabeled nodes from node characteristics and paired associations between subjects. Anirudh and Thiagarajan (2017) proposed a bootstrapped version of graph convolutional neural networks (G-CNNs) that utilized an ensemble of weakly trained G-CNNs and reduced the sensitivity of models on the choice of graph construction [28]. The G-CNNs demonstrated their effectiveness and strong robustness in the noisy graphs. Hu et al. (2021) proposed a learning and interpreting method based on a graph attention network, namely GAT-Li, which included a new graph attention network model and improved the interpretability of the model with feature importance [29]. The model used graph attention layers to learn the node representations and a novel attention pooling layer to obtain the graph representation for functional brain network classification. Li et al. (2021) applied the BrainCNN that contained novel ROI-aware graph convolutional layers and ROI-selection pooling layers for classification [30].

In this study, we aimed to use GNN to perform the classification of young high-functioning ASD and typically developing (TD) individuals based on functional connectome. Although GNNs show a strong learning ability on graph data, a common limitation is the difficulty of interpreting classification results in a way neuroscience can explain [31]. The design of traditional GNNs is mostly based on empirical intuition, heuristics and experimental analysis, of which the representational ability is limited [32]. Here, we analyzed the functional brain network using the Graph Isomorphism Network (GIN) [32], which was proposed as a powerful GNN for graph classification and implement the Weisfeiler-Lehman (WL) graph isomorphism test. The Weisfeiler-Lehman (WL) graph isomorphism test [33,34] is a powerful test known to distinguish a broad class of graphs [35]. In our proposed GIN, the interpretability of the method was improved by adding an attention mechanism to the pooling layer. Different from traditional methods, the proposed Global Attention Pooling method used learnable parameters to obtain the weight of each node and then obtain the graph representation for classification. Furthermore, these weights could also show us which brain regions were the most important in the classification of ASD and typically developing (TD) individuals. Here, we used four independent rs-fMRI datasets of young high-functioning ASD and TD individuals from a public dataset-Autism Brain Imaging Data Exchange (ABIDE) and applied our method as a pilot study.

## 2. Materials and Methods

### 2.1. Materials and Image Preprocessing

We adopted rs-fMRI data of four big datasets (NYU, SDSU, UCLA, and UM) from ABIDE I and ABIDE II [36,37] to reduce irrelevant variance, such as site effects. The dataset we used was preprocessed through our previous study including 130 ASD patients and 173 TD subjects, after strict quality control with visual check, stringent threshold of head motion, and optimized de-noise pipeline [38]. The inclusion criteria is as follows: (a) 10 to 20 years old; (b) IQ  >  70; (c) mean framewise displacement below 0.3 mm; and (d) sufficient quality of anatomical images, assessed by manual review. The details of the preprocessing methods of rs-fMRI data can be found in [38]. The demographic information is shown in Table 1.

**Figure 1:**
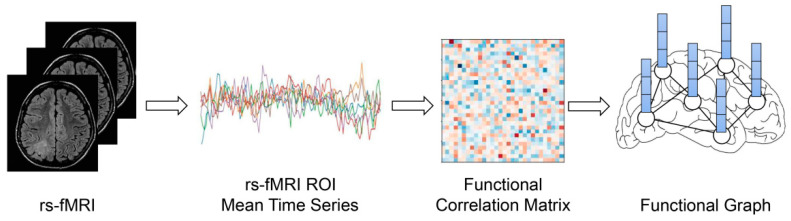
The schematic process of constructing brain FC matrix and functional graph input.

The schematic process of constructing a brain FC network and graphs for model input is shown in Figure 1. The functional connectome for each subject contains 200 cortical ROIs mapped by a widely used parcellation template [38,39]. To be specific, the time series of all voxels were averaged in each ROI, and Pearson’s correlation was calculated between the time series of each pair of 200 ROIs. A 200 × 200 FC matrix for each subject was constructed, which was normalized by Fisher-z transformed correlation coefficients. The group average functional connectome was obtained by averaging the functional connectome of participants in each group.

### 2.2. Proposed Method

**Figure 2:**
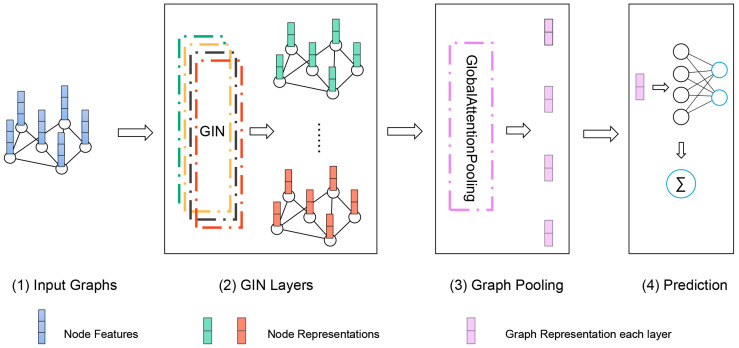
Illustration of our proposed GIN which included four steps, (1) input graphs that corresponds to different subjects; (2) learn node representation through GIN Layers, generating a new representation for each layer; (3) turn the node representations of all layers into the representation of the whole graph by pooling; (4) input graph representation for linear prediction.

We proposed a GIN-based ASD classification method. The architecture of our model is shown in Figure 2. It includes four GIN layers with the node features, edge weights and adjacency matrix as input. The aim of this study was to identify the clinical status of each subject as autism or typically developing individual with the resting-state FC network, which is a graph classification problem. We took the FC network of each subject as a weighted graph with its ROIs as nodes (*N* = 200) and FC as edges. For node features, we selected one hot encoding labels of different ROIs and maintain the disorder. Since the Pearson correlations between each two ROI mean time series represent their functional connectivity, it is more reasonable to consider these values as edge weights rather than node features. As the FC network is a high-dimensional graph with significant redundancy [40], we chose an appropriate threshold M% to reduce the network density. Only the top M percent edges of each FC network were retained in the graph ranked by edge weights.

In our model at *k*-th (*k* = 1,…,4) GIN layer, features $h_{v}^{\left(k\right)}$ of node v were updated to the aggregation of the adjacent node features $h_{u}^{\left(k-1\right)}\left(u\in N\left(v\right)\right)$ of node u multiplied by their respective edge weights $w_{uv}$ and the node embeddings/representations at the input layer or the k-1 GIN layer. Then, the aggregated node features were inputted to the multi-layer perceptron (MLP). We made ϵ a learnable parameter or a fixed scalar. If ϵ was an irrational number, the *k*-th GIN layer made the newly obtained node representations even more injective [32]. Then, GIN updated node representations, as shown in Equation (1).

(1) $$h_{v}^{\left(k\right)}=MLP^{\left(k\right)}\left(\left(1+\epsilon^{\left(k\right)}\right)\cdot h_{v}^{\left(k-1\right)}+\sum_{u\in N\left(v\right)}w_{uv}h_{u}^{\left(k-1\right)}\right)$$

An important aspect of graph-level readout was that the node representation became more refined and global as the number of layers increases. Obtaining information at a deep enough level can improve the discriminative power on the graph classification tasks. However, representations gained from earlier layers may sometimes generalize better [32]. To account for all the structural information, we converted the node embeddings into graph representation through down-sampling after obtaining the node feature representation in GIN at each layer. The outputs of each layer were aggregated in the pooling layer. The common global pooling architecture (Sum, Mean and Max Pooling) do not consider the actual meaning of each node, and tend to eliminate meaningful differences between nodes. Thus, we introduced the attention mechanism here.

The attention mechanism makes it possible to focus more on important features rather than unimportant features [41]. In particular, self-attention, often referred to as internal attention, allowed input features to become the criteria for attention itself [42]. In graph attention pooling, for *k*-th graph $G^{\left(k\right)}$, we assigned a weight $f_{gate}\left(x_{i}^{\left(k\right)}\right)\in R^{d^{\left(k\right)}}\left(i=1,\ldots ,N\right)$ to each node representation $x_{i}^{\left(k\right)}\in R^{d^{\left(k\right)}}(d^{\left(k\right)}\;$ is the *k*-th feature size) obtained from each GIN layer. The value of $f_{gate}\left(x_{i}^{\left(k\right)}\right)$ was initially obtained by linear transformation of the node representation and learned by the softmax function. Next, the weighted sum $g^{\left(k\right)}\in R^{d^{\left(k\right)}}$ of the node embeddings was the representations of $G^{\left(k\right)}$:

(2) $$g^{\left(k\right)}=\overset{N}{\underset{i=1}{\sum }}softmax\left(f_{gate}\left(x_{i}^{\left(k\right)}\right)\right)\cdot x_{i}^{\left(k\right)}$$

Finally, in the READOUT layer, the classification was based on the summarized score of all layers obtained by passing the feature representation of the graph $g^{\left(k\right)}\;$ to the linear layer. That is, the graph representation obtained from each GIN layer contributes to the prediction. The cross entropy was used for the loss function and Adam algorithm as the optimizer.

## 3. Experiment

### 3.1. Experimental Setting

A 10-fold cross-validation strategy was adopted in the experiment. A range of threshold values M (25% to 75%) were applied to obtain a sparse graph, which usually achieved good classification accuracy [43]. After conducting the experiment, we set the threshold value at 25% and trained a GIN with L = 4 hidden layers and M = 2 MLP layers. In addition, we set dropout rate = 0.2 and learning rate = 0.005. Five metrics were used to evaluate the performance of classification between ASD and TD, including Accuracy (ACC), Precision (PRE), Recall (REC), Specificity (SPE) and F1-Score (F1). These metrics are defined as follows.

(3) $$\mathrm{ACC}=\frac{\mathrm{TP}+\mathrm{TN}}{\mathrm{TP}+\mathrm{TN}+\mathrm{FP}+\mathrm{FN}}\;$$

(4) $$\mathrm{PRE}=\frac{\mathrm{TP}}{\mathrm{TP}+\mathrm{FP}}$$

(5) $$\mathrm{REC}=\frac{\mathrm{TP}}{\mathrm{TP}+\mathrm{FN}}$$

(6) $$\mathrm{SPE}=\frac{\mathrm{TN}}{\mathrm{TN}+\mathrm{FP}}$$

(7) $$F1=\frac{2*\mathrm{PRE}*\mathrm{REC}}{\mathrm{PRE}+\mathrm{REC}}$$

TP (True Positive), FP (False Positive), TN (True Negative) and FN (False Negative) could be referred to [44]. ACC represents the percentage of subjects in all samples who obtain the right label. PRE measures the proportion of subjects classified as ASD that are actually ASD. REC measures the proportion of ASD subjects correctly identified among all ASD subjects identified. SPE represents the percentage of subjects in the TD group whose predicted label is correct. F-Measure is the precision and recall weighted harmonic average and is a comprehensive evaluation index. A Higher F-Measure score indicates that the proposed method is effective and the test results are in line with expectations.

### 3.2. Competing Method

We compared our results with a nonlinear classification using SVM, a generalized linear model Logistic Regression and a baseline method (GCN).

(a) Support vector machine (SVM) model with linear kernel and radial basis function (RBF) kernel have been widely used to classify fMRI data for brain diseases [22,45,46].
(b) Logistic regression, a classical machine learning algorithm is often used in dichotomous problems. It has been widely used in the area of epidemiology and medicine, such as looking for the risk factors of a disease.
(c) GCN (Cheby) was used to compare the effectiveness of GIN layer for node representation learning in our model. In this model, we derived node representations from the GCN layer through Chebyshev polynomies [47], and then input the node representation into the same pooling layer and READOUT section of GIN for prediction. The LeakyReLU function was used for the activation function and the cross entropy is used for the loss function.

### 3.3. Results

We evaluated our GIN method and the competing methods on the multi-site ASD detection problem. The results of classification between ASD and TD for five evaluating metrics are shown in Table 2. GIN achieved better and stable results in terms of ACC (mean = 70.63%), PRE (mean = 66.15%), REC (mean = 68.46%), SPE (mean = 72.35%) and F1 (mean = 66.68%). It was noted that the average values of precision and recall for rbf-SVM were 79.13% and 31.54%, which means that the classifier is not good at accurately recognizing the positive samples (ASD samples in this case). In contrast, SVM with linear kernel function had a balanced performance, but was not as good as GIN. The traditional logistic regression did not perform well in five metrics, indicating that ASD identification based on a functional brain network is not a simple linear classification problem. Compared with the GCN(Cheby)-based graph model, the classification performance of our model (with GIN layers) was significantly better in the metrics of ACC, REC and F1. It was suggested that GIN layer, considering graph isomorphism, could improve the classification of ASD from TD based on functional brain networks. Comparing methods of GIN (GIN-0 and GIN-ϵ), we found that GIN-0 was slightly better than GIN-ϵ in each metric. This might be because of its simplicity; GIN-0 has better generalization ability. Similar results were observed in [32]. The results of applying different graph-density thresholds are presented in the Supplementary Materials.

**Figure 3:**
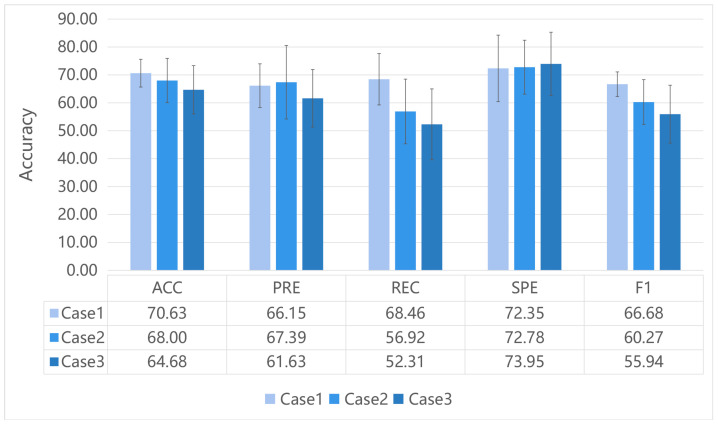
The results of our proposed GIN classification for different ways to construct initial graph. Case1: take the absolute values of FC as edge weights. Case2: keep the edges with positive FC. Case3: keep the original FC values as edge weights.

Figure 3 illustrates the performance of our proposed GIN model for different ways to construct the initial graph. The method of taking the absolute values of FC as edge weights (Case 1) generated the best results in the three metrics (ACC, REC and F1) compared to the other methods. The method of keeping only the positive connectivity of FC as edges (Case 2) and the method of keeping both the positive and negative connectivity of FC as edge weights (Case 3) showed poorer results in almost all the metrics. This suggested that the method of constructing a functional network could affect the classification performance.

### 3.4. Interpretability of GIN

One significant advantage of the GIN model we proposed here was its built-in interpretability. Since the attention mechanism can learn the importance of each node from embeddings, we can interpret the salience of ROIs that provide information for the prediction task at different levels. First, we separated the ASD and TD groups from the training set. For each group, we extracted the weights of the nodes obtained in the last calculation of *k*-th fold after each hidden layer and ranked the ROIs according to their weights. We selected the top salient ROIs (top 50 ROIs, the first quarter of 200 ROIs) $b_{k}^{i}\;\left(k=1,\ldots ,10;i=1,\ldots ,4\right)$ for each fold and each layer. Finally, for *i*-th layer, we took the ROIs $B^{i}\;(i=1,I,4$) that were selected as the top salient ROIs for more than half of the times in the 10-folds (above 5 times) as the final salient ROIs.

**Figure 4:**
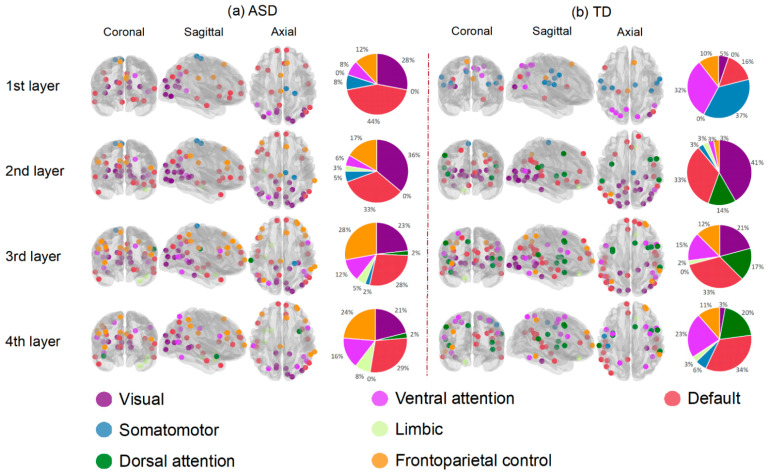
The salient ROIs for classifying ASD and TD in four hidden layers. The dots in the brain template represent salient ROIs in each layer of (**a**) ASD and (**b**) TD group. Different colors correspond to different functional networks. The pie charts showed the proportions of salient ROIs in 7-networks.

Figure 4 displays the salient ROIs associated with ASD and TD, respectively. After neighbor-nodes information was aggregated once, there were few salient ROIs overlapping in both groups. They involved all seven functional networks, including visual, somatomotor, ventral and dorsal attention, limbic, frontoparietal control and default-mode networks [48]. The proportions of salient ROIs in the seven networks are illustrated in the pie charts in Figure 4. More ROIs in the visual and frontoparietal control networks were relevant to ASD. More ROIs in the ventral and dorsal attention networks were associated with TD. The numbers and coordinates of salient ROIs for ASD and typically developing groups are presented in the Supplementary Materials.

## 4. Discussion

In this study, we proposed a graph-based deep learning method to perform the classification task between young high-functioning ASD and typically developing individuals on the functional brain network. Graph neural networks achieve state-of-the-art performance of prediction on neuroimaging data, which take into consideration the network structure of the data. Our proposed GIN method included GIN layers with edge weights information and graph attention pooling layers. Its advantages included being able to capture the local and global connectivity patterns in the functional connectome, as well as the salient ROIs relevant to classification. Compared with the other machine learning and GCN methods, this method showed higher prediction accuracy for ASD classification. GIN improved the average accuracy of ASD classification by 4~6% and the average F1-score by 6~8%. In addition, it outperformed other methods on other metrics, considering the precision and recall rate at the same time. The findings demonstrated that GIN offered a promising way to explore the neuroimaging biomarkers of autism.

Many machine learning methods have been used to perform the classification task of ASD based on functional connectivity. However, these methods usually did not consider the topographic pattern of the whole brain network, which have been found to be altered in ASD [20,22]. Graph Neural Network provides a way to utilize the network structure for classification. GIN is highly expressive of graphs and captures similarities in graph structures. In our experiment, the traditional machine learning methods did not perform well. Although rbf-SVM obtained the highest precision and specificity, it gave the lowest recall rate, which means rbf-SVM had a high false negative rate. This might be because the machine learning methods took the whole brain connectivity as the input features, which may contain a certain level of noise. Too much noise could affect the classification performance [40]. In contrast, GIN considers both the local and global connectivity pattern and is more robust to the noise [32]. GIN is the extension of the WL graph isomorphism test with a high degree of graph expressivity. In the pooling layer, graph representation aggregated node representation from the shallow layer to the deep layer, so the aggregated information was more comprehensive. Dropout was used to improve the generalization ability of the model in the final linear prediction layer. The above results showed that the classification of young high-functioning ASD based on complex brain networks could benefit from deeper and graph-based methods.

A common but important question of using deep learning methods is whether the process can be interpretable. As the salient ROIs in hidden layers could be candidate biomarkers, understanding the salient ROIs associated with the prediction is meaningful for clinical practice. To address this issue, we introduced the attention mechanism into the GIN model. This could provide information about the importance of brain nodes played in the decision making process. As shown in Figure 4, more ROIs belonging to the frontoparietal control network were associated with ASD compared with TD. This is consistent with the existing findings of altered morphometry in the cognitive and affective parts of the frontal cortex in ASD patients [49]. Li et al. (2021) found salient ROIs of frontal gyrus, temporal lobe, cingulate gyrus, occipital pole and angular gyrus for ASD, which corresponded to behavioral deficits in ASD, such as social communication, perception, and execution [30]. Keown et al. (2013) proposed that ASD participants showed local overconnectivity in the visual cortex and local underconnectivity in the prefrontal lobe [50]. Overconnectivity was detected in adolescents with high symptom severity, and mainly in areas involved in visual processing [50]. Existing evidence suggested that vision may play an important role in the neuropsychological profile of ASD [51]. The underconnectivity in the frontal region can be associated with impaired executive function and cognitive control in ASD [50,52]. In addition, studies on the structural network also found similar regions to be altered in ASD. Liloia et al. (2021) found middle occipital gyrus in GM co-alteration network of ASD [53]. Wang et al. (2021) used the deep learning method and structural covariance network to classify ASD and found salient ROIs in prefrontal regions [54]. In our experiment, each layer had salient ROIs from the visual network associated with ASD, and more ROIs of the frontoparietal control network became more significant with the depth of layers. These two networks are the neurobiological basis of visual attention, which is impaired in ASD. Thus, these networks may be responsible for the social-cognitive deficits in ASD. The salient ROIs selected can be used as biomarkers to identify ASD and TD in this study, but further studies are needed to test its generalizability.

In addition, this study also demonstrated that the method of constructing the initial graph could affect the classification performance. Functional connectome is a special network, as it is not a physical network but rather a statistic network. Although there is no established explanation for the negative correlation between brain regions, it would lose a lot of the information to only use the positive FC to construct the initial graph. Many studies have evaluated different ways to construct functional networks [29,30,55]; however, it is unclear how it would affect the performance of deep learning. In the process of constructing the functional graph from FC, we defined the weight of the edge as the connection strength. Considering the definition of Pearson’s correlation coefficient, the absolute value of it denoted the strength of the connection. Weak connections have been proposed to lead to less stable results [43,56]. There were a large number of weak connections in case 2 and negative weights in case 3 in our experiment, which may have weakened the connection strength of the graph. From the experimental results, we could also find poor performance of all metrics in case 3. Further studies are needed to explore whether the positive and negative of correlation can be applied to input graphs.

### Limitation and Future Work

We proposed a method from GNN to utilize functional connectome to identify ASD from typically developing individuals and achieved good performance. However, there are several limitations to be noted. First of all, the current study focused only on identifying young high-functioning ASD. In the future, the method should be tested on the more general ASD population. Secondly, from the perspective of deep learning, the dataset used in this work is relatively small and the performance scores are not very high. Considering the difficulty of acquiring neuroimaging data, especially for clinical data, it is difficult to acquire data on the same scale as computer vision. As it is a common issue in neuroimaging studies, a solution might be to refine the algorithm and improve the quality of the data itself. In this study, we chose the datasets with large data size from the multi-sites ABIDE dataset to reduce the sites variability and preprocessed the data with stringent criterion for quality-control. We also tried different ways to sparse the graph and to construct the graph to reduce the noise affect. In addition, the main focus of this study was the application of the model incorporating deep learning and functional connectome. Compared to other methods, this pilot study demonstrated a better performance. The next step may be to use more data samples and to utilize more feature information to enrich ROI information, such as using multi-modal data. We hope to improve the classification performance of ASD by applying more advanced deep learning techniques. Thirdly, our method was only applied to a high-functioning autism group because high-functioning and low-functioning groups differ in functional connectivity [57]. Our method should be tested on low-functioning groups in the future. In the future, we will further improve the training model and incorporate the evaluation of the importance of the edges. Lastly, although it showed good performance on the classification, using neuroimaging as biomarkers for diagnosis or treatment proved to be inadequate. Combining more types of biomarkers, such as genetics, may benefit diagnosis and treatment.

## 5. Conclusions

We proposed an improved GIN model for young high-functioning ASD identification based on functional connectome. We also evaluated the importance of each node in the classification to improve the interpretability of the algorithm. The proposed GIN performed better than common machine learning methods in ASD identification. The most salient regions identified by the model involved visual network, frontoparietal control network and default mode network, which could be potential biomarkers for ASD detection. Our method could be applied in studying other neurodevelopmental disorders and promised to improve clinical diagnosis in the future.

## Figures and Tables

**Table 1:** The demographic information of our datasets.

	ASD			TD	
Site	Age (Mean ± S.D.)	Sex (M/F)	ADOS (Mean ± S.D.)	Age (Mean ± S.D.)	Sex (M/F)
NYU	13.34 ± 2.62	31/5	11.72 ± 4.01	13.71 ± 2.56	45/11
SDSU	14.50 ± 2.53	29/4	12.88 ± 4.73	14.35 ± 2.15	25/6
UCLA	14.07 ± 2.23	27/1	11.78 ± 3.77	13.55 ± 1.67	25/7
UM	14.72 ± 1.83	28/5	11.44 ± 5.16	15.00 ± 2.49	41/13

**Table 2:** Classification performance of five methods.

	Rbf-SVM	Linear-SVM	Logistic	GCN (Cheby)	GIN- ϵ (0.1)	GIN-0
Accuracy (%, mean ± S.D.)	67.42 ± 3.55	67.42 ± 9.55	65.48 ± 10.43	66.38 ± 10.76	68.00 ± 10.51	**70.63 ± 4.97**
Precision (%, mean ± S.D.)	**79.13 ± 12.73**	60.37 ± 11.54	58.50 ± 12.80	66.16 ± 19.17	63.60 ± 13.08	66.15 ± 7.83
Recall (%, mean ± S.D.)	31.54 ± 7.65	61.54 ± 18.13	59.23 ± 17.03	53.85 ± 11.47	66.15 ± 7.43	**68.46 ± 9.21**
Specificity (%, mean ± S.D.)	**93.33 ± 5.11**	71.67 ± 7.61	70.00 ± 9.87	75.85 ± 17.36	69.31 ± 15.84	72.35 ± 11.90
F1-score (%, mean ± S.D.)	44.42 ± 7.84	60.48 ± 14.12	58.44 ± 14.21	58.23 ± 11.23	64.43 ± 9.50	**66.68 ± 4.40**

The best results were shown in bold. ϵ is the learnable parameter, here we set the initial value to 0.1. GIN-0 means we do not set ϵ .

## Data Availability

All data is available from the ABIDE repository (https://fcon_1000.projects.nitrc.org/indi/abide/, accessed on 1 March 2019).

## Supplementary Materials

The following supporting information can be downloaded at: https://www.mdpi.com/article/10.3390/brainsci12070883/s1, Figure S1: The classification accuracy of our proposed GIN model with different threshold values. Table S1: The coordinates of salient ROIs for ASD group in four hidden layers. Table S2: The coordinates of salient ROIs for TD group in four hidden layers. Table S3. Description of ROI’s abbreviation.
