# Supplementary material for: Identification of Young High-Functioning Autism Individuals Based on Functional Connectome Using Graph Isomorphism Network: A Pilot Study
Source: Brain Sci. 2022 Jul 5;12(7):883. doi: 10.3390/brainsci12070883 (PMC9315722; doi:10.3390/brainsci12070883)
Supplement: Supplementary file 1 [file brainsci-12-00883-s001.zip › brainsci-1770201-supplementary.pdf]

brain sciences

MDPI

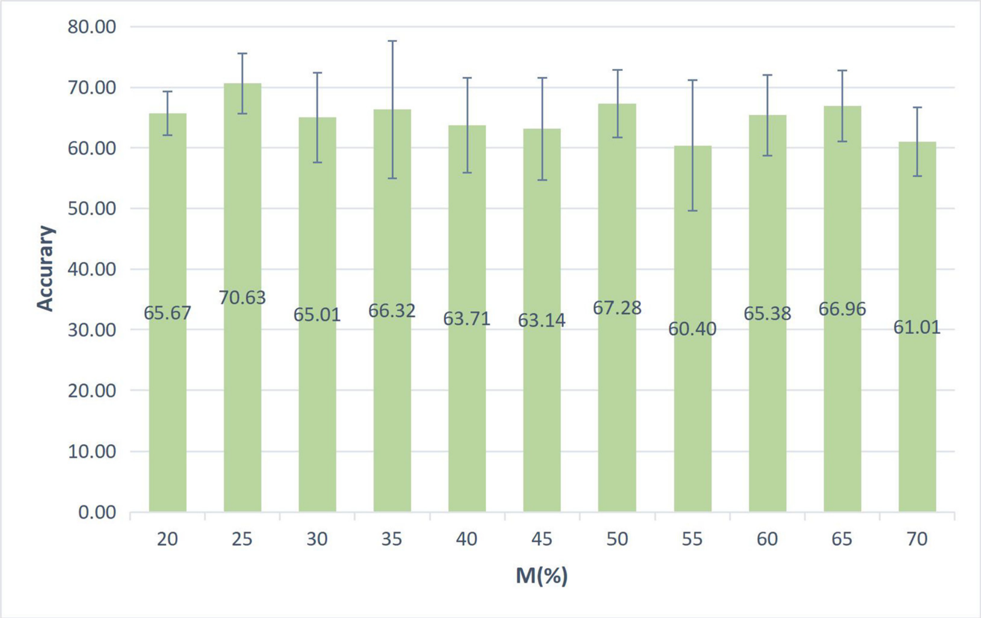


**Figure S1.** The classification accuracy of our proposed GIN model with different threshold values.

**Table S1.** The coordinates of salient ROIs for ASD group in four hidden layers.

| ASD | | | | | | | | |
|---|---|---|---|---|---|---|---|---|
| NO. | 1st layer | | 2nd layer | | 3rd layer | | 4th layer | |
| | ROI | Coordinates | ROI | Coordinates | ROI | Coordinates | ROI | Coordinates |
| 1 | LH_Default_PHC_1 | (-26, -32, -18) | LH_Vis_6 | (-14, -44, -2) | RH_Cont_PFCl_4 | (46, 24, 26) | LH_Vis_6 | (-14, -44, -2) |
| 2 | RH_Cont_PFCmp_2 | (8, 24, 56) | LH_DorsAttn_Post_3 | (-26, -70, 38) | RH_Cont_PFCmp_1 | (8, 30, 28) | RH_Cont_PFCl_4 | (46, 24, 26) |
| 3 | LH_Cont_Cing_1 | (-4, -28, 28) | LH_Default_PFC_5 | (-52, 22, 8) | RH_Cont_Par_1 | (62, -38, 36) | LH_Limbic_TempPole_4 | (-42, 8, -18) |
| 4 | LH_Default_PFC_1 | (-34, 20, -14) | LH_Default_pCunPCC_4 | (-6, -54, 42) | RH_Default_Par_3 | (50, -58, 44) | RH_Vis_9 | (8, -76, 8) |
| 5 | LH_Default_PFC_4 | (-12, 64, -6) | LH_Default_PHC_1 | (-26, -32, -18) | RH_Default_Temp_1 | (48, 12, -30) | RH_Vis_11 | (42, -80, 10) |
| 6 | LH_Default_PFC_5 | (-52, 22, 8) | RH_SomMot_19 | (20, -30, 70) | LH_Vis_6 | (-14, -44, -2) | RH_DorsAttn_Post_1 | (50, -54, -16) |
| 7 | LH_Default_pCunPCC_4 | (-6, -54, 42) | RH_Cont_PFCl_4 | (46, 24, 26) | LH_Limbic_TempPole_2 | (-44, -20, -30) | RH_DorsAttn_Post_2 | (52, -60, 10) |
| 8 | RH_SomMot_19 | (20, -30, 70) | LH_Limbic_TempPole_2 | (-44, -20, -30) | LH_Cont_Cing_1 | (-4, -28, 28) | RH_SalVentAttn_FrOperIns_1 | (40, 6, -16) |
| 9 | RH_Default_Par_1 | (46, -70, 28) | LH_Cont_Cing_1 | (-4, -28, 28) | LH_Default_Temp_2 | (-60, -18, -22) | RH_Cont_Par_2 | (52, -42, 48) |
| 10 | RH_Default_Par_3 | (50, -58, 44) | LH_Default_PFC_4 | (-12, 64, -6) | LH_Default_PFC_5 | (-52, 22, 8) | RH_Cont_PFCl_2 | (30, 58, 4) |
| 11 | LH_DorsAttn_Post_3 | (-26, -70, 38) | RH_Vis_6 | (12, -92, -6) | RH_Vis_6 | (12, -92, -6) | RH_Default_Par_3 | (50, -58, 44) |
| 12 | RH_Vis_6 | (12, -92, -6) | RH_Default_Par_3 | (50, -58, 44) | RH_DorsAttn_Post_2 | (52, -60, 10) | RH_Default_Temp_2 | (60, -12, -20) |
| 13 | RH_Default_PFCv_1 | (50, 28, 0) | LH_DorsAttn_Post_2 | (-56, -60, -2) | LH_Vis_12 | (-12, -72, 22) | LH_Vis_12 | (-12, -72, 22) |
| 14 | RH_Default_PFCdPFCm_1 | (4, 36, -14) | LH_Cont_PFCl_3 | (-42, 40, 16) | LH_DorsAttn_Post_3 | (-26, -70, 38) | LH_DorsAttn_Post_4 | (-54, -26, 42) |
| 15 | LH_Vis_10 | (-12, -70, 8) | LH_Default_PFC_1 | (-34, 20, -14) | LH_SalVentAttn_ParOper_2 | (-62, -26, 28) | LH_Limbic_TempPole_2 | (-44, -20, -30) |
| 16 | LH_Vis_11 | (-40, -84, 10) | RH_Vis_9 | (8, -76, 8) | LH_Limbic_TempPole_3 | (-28, 10, -34) | LH_Limbic_TempPole_3 | (-28, 10, -34) |
| 17 | LH_Vis_12 | (-12, -72, 22) | RH_Vis_12 | (20, -90, 22) | LH_Cont_PFCl_3 | (-42, 40, 16) | LH_Cont_PFCl_3 | (-42, 40, 16) |
| 18 | LH_DorsAttn_Post_2 | (-56, -60, -2) | RH_Cont_PFCmp_2 | (8, 24, 56) | LH_Default_Par_1 | (-48, -58, 18) | LH_Cont_Cing_1 | (-4, -28, 28) |
| 19 | LH_Cont_Cing_2 | (-4, 4, 30) | RH_Default_Par_1 | (46, -70, 28) | LH_Default_PHC_1 | (-26, -32, -18) | LH_Default_Temp_2 | (-60, -18, -22) |

| | | | | | | | | |
|---|---|---|---|---|---|---|---|---|
| **20** | RH_Vis_5 | (48, -72, -6) | LH_Vis_10 | (-12, -70, 8) | RH_Vis_9 | (8, -76, 8) | LH_Default_Par_1 | (-48, -58, 18) |
| **21** | RH_Vis_11 | (42, -80, 10) | LH_Vis_12 | (-12, -72, 22) | RH_Vis_12 | (20, -90, 22) | LH_Default_PFC_5 | (-52, 22, 8) |
| **22** | RH_Vis_12 | (20, -90, 22) | RH_Vis_5 | (48, -72, -6) | RH_SomMot_19 | (20, -30, 70) | LH_Default_PFC_7 | (-8, 60, 20) |
| **23** | RH_SomMot_18 | (6, -22, 68) | RH_Vis_11 | (42, -80, 10) | RH_DorsAttn_Post_1 | (50, -54, -16) | RH_Vis_12 | (20, -90, 22) |
| **24** | RH_Default_Temp_2 | (60, -12, -20) | RH_SomMot_18 | (6, -22, 68) | RH_Cont_Par_2 | (52, -42, 48) | RH_DorsAttn_Post_8 | (26, -62, 58) |
| **25** | RH_Default_PFCdPFCm_4 | (8, 58, 18) | RH_Cont_Par_1 | (62, -38, 36) | RH_Cont_PFCl_5 | (30, 48, 28) | RH_Cont_Par_1 | (62, -38, 36) |
| **26** | | | RH_Cont_PFCmp_1 | (8, 30, 28) | RH_Cont_PFCl_6 | (40, 34, 38) | RH_Cont_PFCl_5 | (30, 48, 28) |
| **27** | | | RH_Default_PFCv_1 | (50, 28, 0) | LH_Vis_8 | (-48, -70, 10) | RH_Cont_PFCmp_2 | (8, 24, 56) |
| **28** | | | LH_Vis_3 | (-46, -70, -8) | LH_Vis_10 | (-12, -70, 8) | RH_Default_Par_1 | (46, -70, 28) |
| **29** | | | LH_Vis_5 | (-26, -96, -12) | LH_DorsAttn_Post_2 | (-56, -60, -2) | RH_Default_PFCdPFCm_4 | (8, 58, 18) |
| **30** | | | LH_Vis_8 | (-48, -70, 10) | LH_Cont_Par_2 | (-34, -62, 48) | LH_Vis_10 | (-12, -70, 8) |
| **31** | | | LH_Default_Temp_2 | (-60, -18, -22) | LH_Cont_PFCl_4 | (-44, 20, 28) | LH_DorsAttn_Post_3 | (-26, -70, 38) |
| **32** | | | LH_Default_PFC_3 | (-46, 30, -8) | LH_Default_Temp_3 | (-56, -6, -12) | LH_Default_Temp_4 | (-58, -30, -4) |
| **33** | | | RH_Vis_7 | (16, -46, -2) | LH_Default_PFC_4 | (-12, 64, -6) | RH_Vis_6 | (12, -92, -6) |
| **34** | | | RH_Vis_10 | (22, -60, 8) | LH_Default_pCunPCC_4 | (-6, -54, 42) | RH_Vis_7 | (16, -46, -2) |
| **35** | | | RH_Default_Temp_1 | (48, 12, -30) | RH_Vis_1 | (38, -34, -24) | RH_DorsAttn_PrCv_1 | (52, 10, 20) |
| **36** | | | RH_Default_PFCdPFCm_4 | (8, 58, 18) | RH_Vis_7 | (16, -46, -2) | RH_Cont_PFCmp_1 | (8, 30, 28) |
| **37** | | | | | RH_Vis_11 | (42, -80, 10) | RH_Default_Temp_1 | (48, 12, -30) |
| **38** | | | | | RH_DorsAttn_PrCv_1 | (52, 10, 20) | RH_Default_PFCdPFCm_2 | (8, 42, 4) |
| **39** | | | | | RH_Cont_PFCl_2 | (30, 58, 4) | | |
| **40** | | | | | RH_Cont_PFCmp_2 | (8, 24, 56) | | |
| **41** | | | | | RH_Default_Par_1 | (46, -70, 28) | | |
| **42** | | | | | RH_Default_Temp_2 | (60, -12, -20) | | |
| **43** | | | | | RH_Default_PFCdPFCm_4 | (8, 58, 18) | | |

for each layer, we took the ROIs that selected as the top salient ROIs for more than half of the times in the 10-folds (above 5 times) as the final salient ROIs. So the number of salient ROIs varied for each layer.

**Table S2.** The serial numbers and coordinates of salient ROIs for TD group in four hidden layers.

| TD | | | | | | | | |
|---|---|---|---|---|---|---|---|---|
| **NO.** | **1st layer** | | **2nd layer** | | **3rd layer** | | **4th layer** | |
| | **ROI** | **Coordinates** | **ROI** | **Coordinates** | **ROI** | **Coordinates** | **ROI** | **Coordinates** |
| **1** | LH_Default_PHC_1 | (-26, -32, -18) | LH_Default_pCunPCC_2 | (-6, -54, 28) | LH_Default_Temp_4 | (-58, -30, -4) | LH_DorsAttn_PrCv_1 | (-48, 6, 28) |
| **2** | RH_Cont_PFCmp_2 | (8, 24, 56) | RH_Default_PFCdPFCm_5 | (16, 46, 44) | LH_Default_Par_3 | (-58, -54, 28) | LH_SalVentAttn_ParOper_2 | (-62, -26, 28) |
| **3** | RH_Default_Par_1 | (46, -70, 28) | LH_Vis_1 | (-24, -54, -10) | LH_Default_Par_4 | (-46, -66, 38) | LH_SalVentAttn_FrOperIns_1 | (-40, -4, -4) |
| **4** | LH_SomMot_5 | (-52, -22, 18) | RH_Vis_1 | (38, -34, -24) | LH_Default_PFC_6 | (-6, 44, 8) | LH_SalVentAttn_FrOperIns_3 | (-38, 0, 10) |
| **5** | LH_DorsAttn_Post_3 | (-26, -70, 38) | LH_Vis_2 | (-26, -78, -14) | LH_Default_PFC_7 | (-8, 60, 20) | LH_Default_Temp_2 | (-60, -18, -22) |
| **6** | LH_DorsAttn_Post_7 | (-16, -72, 54) | LH_Vis_5 | (-26, -96, -12) | LH_Default_pCunPCC_2 | (-6, -54, 28) | LH_Default_Temp_4 | (-58, -30, -4) |

| | | | | | | | | |
|---|---|---|---|---|---|---|---|---|
| **7** | LH_DorsAttn_Post_8 | (-30, -60, 60) | LH_Vis_11 | (-40, -84, 10) | RH_SalVentAttn_PrC_1 | (50, 4, 40) | LH_Default_Par_1 | (-48, -58, 18) |
| **8** | LH_Cont_Cing_1 | (-4, -28, 28) | LH_Default_Temp_5 | (-58, -42, 8) | RH_Limbic_OFC_1 | (12, 38, -22) | LH_Default_Par_3 | (-58, -54, 28) |
| **9** | LH_Default_PFC_5 | (-52, 22, 8) | LH_Default_Par_3 | (-58, -54, 28) | RH_Default_Temp_3 | (54, -6, -10) | LH_Default_PFC_6 | (-6, 44, 8) |
| **10** | RH_SomMot_7 | (58, -4, 30) | LH_Default_Par_4 | (-46, -66, 38) | RH_Default_Temp_5 | (52, -32, 2) | LH_Default_PFC_7 | (-8, 60, 20) |
| **11** | RH_SomMot_8 | (10, -16, 42) | LH_Default_PFC_6 | (-6, 44, 8) | LH_SalVentAttn_FrOperIns_4 | (-52, 8, 10) | RH_DorsAttn_Post_1 | (50, -54, -16) |
| **12** | LH_SomMot_3 | (-36, -22, 16) | RH_Vis_5 | (48, -72, -6) | LH_Default_Temp_2 | (-60, -18, -22) | RH_DorsAttn_FEF_2 | (26, 8, 58) |
| **13** | LH_SomMot_6 | (-56, -8, 30) | RH_Vis_8 | (30, -94, -4) | LH_Default_Temp_5 | (-58, -42, 8) | RH_SalVentAttn_PrC_1 | (50, 4, 40) |
| **14** | LH_DorsAttn_Post_9 | (-6, -60, 56) | RH_SalVentAttn_PrC_1 | (50, 4, 40) | LH_Default_Par_1 | (-48, -58, 18) | RH_SalVentAttn_Med_1 | (8, 10, 42) |
| **15** | RH_Vis_11 | (42, -80, 10) | RH_Limbic_OFC_1 | (12, 38, -22) | LH_Default_Par_2 | (-38, -80, 32) | RH_Cont_PFCl_2 | (30, 58, 4) |
| **16** | RH_SomMot_4 | (44, -28, 18) | RH_Default_Temp_3 | (54, -6, -10) | RH_Vis_1 | (38, -34, -24) | RH_Default_Temp_2 | (60, -12, -20) |
| **17** | RH_SomMot_6 | (56, -12, 14) | LH_Vis_7 | (-6, -92, -4) | RH_Cont_PFCl_2 | (30, 58, 4) | RH_Default_Temp_5 | (52, -32, 2) |
| **18** | RH_DorsAttn_Post_2 | (52, -60, 10) | LH_Vis_9 | (-22, -96, 6) | RH_Default_Temp_2 | (60, -12, -20) | LH_SomMot_7 | (-48, -8, 46) |
| **19** | RH_DorsAttn_Post_6 | (14, -74, 52) | LH_SalVentAttn_ParOper_1 | (-56, -40, 20) | RH_Default_PFCdPFCm_5 | (16, 46, 44) | LH_SomMot_10 | (-40, -26, 56) |
| **20** | | | LH_Default_Par_2 | (-38, -80, 32) | LH_Vis_5 | (-26, -96, -12) | LH_DorsAttn_Post_10 | (-18, -54, 68) |
| **21** | | | RH_SomMot_18 | (6, -22, 68) | LH_Vis_7 | (-6, -92, -4) | LH_DorsAttn_FEF_1 | (-32, -4, 54) |
| **22** | | | LH_Vis_4 | (-10, -68, -4) | LH_Cont_Par_2 | (-34, -62, 48) | LH_Default_Temp_5 | (-58, -42, 8) |
| **23** | | | LH_Vis_6 | (-14, -44, -2) | RH_Vis_5 | (48, -72, -6) | LH_Default_Par_2 | (-38, -80, 32) |
| **24** | | | LH_SalVentAttn_FrOperIns_1 | (-40, -4, -4) | RH_Vis_7 | (16, -46, -2) | LH_Default_Par_4 | (-46, -66, 38) |
| **25** | | | LH_Cont_Par_2 | (-34, -62, 48) | RH_Vis_8 | (30, -94, -4) | LH_Default_pCunPCC_2 | (-6, -54, 28) |
| **26** | | | LH_Default_PFC_1 | (-34, 20, -14) | RH_DorsAttn_Post_1 | (50, -54, -16) | RH_Vis_1 | (38, -34, -24) |
| **27** | | | RH_Vis_4 | (12, -64, -4) | RH_DorsAttn_Post_2 | (52, -60, 10) | RH_DorsAttn_Post_8 | (26, -62, 58) |
| **28** | | | RH_Vis_7 | (16, -46, -2) | RH_DorsAttn_FEF_2 | (26, 8, 58) | RH_DorsAttn_FEF_1 | (34, -4, 52) |
| **29** | | | RH_Vis_10 | (22, -60, 8) | RH_SalVentAttn_TempOccPar_2 | (60, -38, 16) | RH_DorsAttn_PrCv_1 | (52, 10, 20) |
| **30** | | | RH_Vis_11 | (42, -80, 10) | RH_SalVentAttn_FrOperIns_1 | (40, 6, -16) | RH_SalVentAttn_TempOccPar_2 | (60, -38, 16) |
| **31** | | | RH_DorsAttn_Post_2 | (52, -60, 10) | RH_SalVentAttn_FrOperIns_4 | (44, 6, 4) | RH_SalVentAttn_FrOperIns_4 | (44, 6, 4) |
| **32** | | | RH_SalVentAttn_TempOccPar_2 | (60, -38, 16) | RH_Cont_Temp_1 | (62, -42, -12) | RH_Limbic_OFC_1 | (12, 38, -22) |
| **33** | | | RH_SalVentAttn_FrOperIns_4 | (44, 6, 4) | RH_Cont_PFCl_5 | (30, 48, 28) | RH_Cont_Par_2 | (52, -42, 48) |
| **34** | | | RH_Default_Par_2 | (54, -50, 28) | RH_Default_Par_1 | (46, -70, 28) | RH_Cont_Temp_1 | (62, -42, -12) |
| **35** | | | RH_Default_Temp_5 | (52, -32, 2) | LH_Vis_1 | (-24, -54, -10) | RH_Cont_PFCl_5 | (30, 48, 28) |
| **36** | | | RH_Default_PFCdPFCm_1 | (4, 36, -14) | LH_Vis_8 | (-48, -70, 10) | | |
| **37** | | | | | LH_Vis_11 | (-40, -84, 10) | | |

| | | | | | | | | |
|---|---|---|---|---|---|---|---|---|
| 38 | | | | | LH_DorsAttn_Post_2 | (-56, -60, -2) | | |
| 39 | | | | | LH_DorsAttn_Post_4 | (-54, -26, 42) | | |
| 40 | | | | | LH_DorsAttn_PrCv_1 | (-48, 6, 28) | | |
| 41 | | | | | LH_SalVentAttn_FrOperIns_1 | (-40, -4, -4) | | |
| 42 | | | | | LH_SalVentAttn_FrOperIns_3 | (-38, 0, 10) | | |
| 43 | | | | | RH_Vis_11 | (42, -80, 10) | | |
| 44 | | | | | RH_DorsAttn_PrCv_1 | (52, 10, 20) | | |
| 45 | | | | | RH_SalVentAttn_Med_1 | (8, 10, 42) | | |
| 46 | | | | | RH_Cont_Par_2 | (52, -42, 48) | | |
| 47 | | | | | RH_Cont_Par_3 | (38, -62, 48) | | |
| 48 | | | | | RH_Default_PFCdPFCm_4 | (8, 58, 18) | | |

for each layer, we took the ROIs that selected as the top salient ROIs for more than half of the times in the 10-folds (above 5 times) as the final salient ROIs. So the number of salient ROIs varied for each layer.

**Table S3.** Description of ROI's abbreviation.

| ROI name | Network name | Full component name |
|---|---|---|
| LH_Vis | visual | visual |
| LH_SomMot | somatomotor | somatomotor |
| LH_DorsAttn_Post | dorsal attention | posterior |
| LH_DorsAttn_FEF | dorsal attention | frontal eye fields |
| LH_DorsAttn_PrCv | dorsal attention | precentral ventral |
| LH_SalVentAttn_ParOper | salience/ventral attention | parietal operculum |
| LH_SalVentAttn_TempOcc | salience/ventral attention | temporal occipital |
| LH_SalVentAttn_FrOperIns | salience/ventral attention | frontal operculum insula |
| LH_SalVentAttn_PFCl | salience/ventral attention | lateral prefrontal cortex |
| LH_SalVentAttn_Med | salience/ventral attention | medial |
| LH_Limbic_OFC | limbic | orbital frontal cortex |
| LH_Limbic_TempPole | limbic | temporal pole |
| LH_Cont_Par | control | parietal |
| LH_Cont_Temp | control | temporal |
| LH_Cont_PFCd | control | dorsal prefrontal cortex |
| LH_Cont_PFCl | control | lateral prefrontal cortex |
| LH_Cont_OFC | control | orbital frontal cortex |
| LH_Cont_PFCv | control | ventral prefrontal cortex |
| LH_Cont_pCun | control | precuneus |
| LH_Cont_Cing | control | cingulate |
| LH_Cont_PFCmp | control | medial posterior prefrontal cortex |
| LH_Default_Par | default | parietal |
| LH_Default_Temp | default | temporal |
| LH_Default_PFC | default | prefrontal cortex |
| LH_Default_pCunPCC | default | precuneus posterior cingulate cortex |

| | | |
|---|---|---|
| LH_Default_PHC | default | parahippocampal cortex |
| RH_Vis | visual | visual |
| RH_SomMot | somatomotor | somatomotor |
| RH_DorsAttn_Post | dorsal attention | posterior |
| RH_DorsAttn_FEF | dorsal attention | frontal eye fields |
| RH_DorsAttn_PrCv | dorsal attention | precentral ventral |
| RH_SalVentAttn_TempOccPar | salience/ventral attention | temporal occipital parietal |
| RH_SalVentAttn_PrC | salience/ventral attention | precentral |
| RH_SalVentAttn_FrOperIns | salience/ventral attention | frontal operculum insula |
| RH_SalVentAttn_PFCv | salience/ventral attention | ventral prefrontal cortex |
| RH_SalVentAttn_PFCl | salience/ventral attention | lateral prefrontal cortex |
| RH_SalVentAttn_Med | salience/ventral attention | medial |
| RH_Limbic_OFC | limbic | orbital frontal cortex |
| RH_Limbic_TempPole | limbic | temporal pole |
| RH_Cont_Par | control | parietal |
| RH_Cont_Temp | control | temporal |
| RH_Cont_PFCv | control | ventral prefrontal cortex |
| RH_Cont_PFCl | control | lateral prefrontal cortex |
| RH_Cont_pCun | control | precuneus |
| RH_Cont_Cing | control | cingulate |
| RH_Cont_PFCmp | control | medial posterior prefrontal cortex |
| RH_Default_Par | default | parietal |
| RH_Default_Temp | default | temporal |
| RH_Default_PFCv | default | ventral prefrontal cortex |
| RH_Default_PFCdPFCm | default | dorsal prefrontal cortex medial prefrontal cortex |
| RH_Default_pCunPCC | default | precuneus posterior cingulate cortex |

LH represents the left hemisphere and RH represents the right hemisphere of the brain.
